# Older Age and High Serum Ferritin Levels Associated With the Risk of Chronic Cytopenia in Hemodialysis Patients

**DOI:** 10.3389/fmed.2020.568350

**Published:** 2020-10-29

**Authors:** Yu-Ting Lee, Wei-Yu Wang, Chin-Ho Kuo, Ming-Yang Lee, Yin-Che Lu, Chih-Yen Hsiao, Yueh-Han Hsu, Peir-Haur Hung

**Affiliations:** ^1^Division of Hematology and Oncology, Department of Medicine, Ditmanson Medical Foundation Chia-Yi Christian Hospital, Chiayi, Taiwan; ^2^Min-Hwei Junior College of Health Care Management, Tainan, Taiwan; ^3^Division of Nephrology, Department of Medicine, Ditmanson Medical Foundation Chia-Yi Christian Hospital, Chiayi, Taiwan; ^4^Department of Hospital and Health Care Administration, Chia Nan University of Pharmacy and Science, Tainan, Taiwan; ^5^Department of Applied Life Science and Health, Chia Nan University of Pharmacy and Science, Tainan, Taiwan

**Keywords:** end stage renal disease, leucopenia, thrombocytopenia, hemodialysis, risk factors

## Abstract

Leukopenia or thrombocytopenia is sometimes observed in end-stage renal disease (ESRD) patients, but the association between chronic leukopenia or thrombocytopenia and hemodialysis (HD) is still unclear. We aimed to investigate the incidence of chronic leukopenia or thrombocytopenia in patients with ESRD who received HD and to determine the risk factors of this complication. We retrospectively analyzed ESRD patients treated with HD at Ditmanson Medical Foundation Chia-Yi Christian Hospital in 2018. The risk factors for the occurrence of chronic leukopenia and thrombocytopenia were analyzed by Cox regression models. Of the 473 patients in our study cohort, 46 (9.7%) patients had a hematologic abnormality, including 18 patients with chronic leukopenia, 18 with chronic thrombocytopenia, and 10 with pancytopenia. Multivariate analysis revealed that patient age ≥60 years at the initiation of dialysis was a significant predictor for both chronic leukopenia [adjusted hazard ratio (aHR), 2.71; 95% confidence interval (CI), 1.06-6.89] and chronic thrombocytopenia (aHR, 2.83; 95% CI, 1.08–7.35). Chronic liver disease (aHR, 3.31; 95% CI, 1.27–8.61) and serum ferritin levels >800 mg/dl (aHR, 3.29; 95% CI, 1.29–8.39) were risk factors for chronic thrombocytopenia. A trend showed that vitamin D from intravenous supplementation (aHR, 0.13; 95% CI, 0.01–1.16, *P* = 0.066) and serum phosphorous level (aHR, 0.73; 95% CI, 0.53–1.02, *P* = 0.068) may be associated with chronic thrombocytopenia. Our study demonstrated that hematological abnormality was a long-term complication of HD. These results reveal that older patients with HD and high serum ferritin levels are at an elevated risk for chronic cytopenia. Healthcare professionals should be aware of this risk when treating HD patients in order to improve their prognosis.

## Introduction

Taiwan has one of the highest prevalence rates of end-stage renal disease (ESRD) in the world (1). In 2018, the incidence rate of dialysis was 493 patients per million people in the general population in Taiwan (2–4).

The life expectancy of ESRD patients has been greatly improved by multidisciplinary patient education and high-quality care (2, 5). Long-term complications affect patients' quality of life. Dialysis is associated with complications such as anemia, secondary hyperparathyroidism, and bone disorder (6). Anemia is a well-known hematological problem in chronic kidney disease. The use of erythropoiesis-stimulating agents increases hemoglobin concentrations and improves patient-perceived quality of life (7).

In contrast to anemia, other hematologic abnormalities are less explored (8–11). Leukopenia and thrombocytopenia are observed temporarily at the initiation of dialysis therapy and are usually associated with dialyzer membranes and activation of the complement system (9, 12, 13). The cause of cytopenia is complex. For example, platelet may be consumed between blood and artificial surfaces. Malnutrition, not uncommon in ESRD, may probably suppress hematopoiesis (14, 15). Chronic cytopenia and bone marrow fibrosis directed by secondary hyperparathyroidism have been reported (8, 10, 16–18); however, the prevalence of chronic leukopenia and thrombocytopenia is poorly understood. Therefore, we developed this retrospective study to identify the risk factors and incidence of chronic leukopenia and chronic thrombocytopenia in patients with ESRD.

## Materials and Methods

### Study Population

Patients treated with hemodialysis (HD) at Ditmanson Medical Foundation Chia-Yi Christian Hospital from January 1, 2018, to December 31, 2018, were enrolled in this study. Patients with chronic leukopenia and thrombocytopenia before or on regular HD for <6 months were excluded in order to eliminate the impact of early mortality (19, 20). This retrospective study was conducted in concordance with institutional patient safety laws and the Declaration of Helsinki and was duly approved by the institutional review board of Ditmanson Medical Foundation Chia-Yi Christian Hospital (CYCH-IRB- 2019042).

### Definition of Leukopenia and Thrombocytopenia

Participants' clinical information [including age, gender, body mass index (BMI), vitamin D from intravenous (IV) supplementation, and iron supplementation], comorbidities [including hepatitis C virus (HCV), hepatitis B virus (HBV), chronic liver disease, rheumatologic disease, diabetes mellitus (DM), cerebral vascular disease, hypertension, and cancer], laboratory data [complete blood count (CBC), white blood cell count (WBC), platelet (PLT) counts, C-reactive protein, phosphate, ferritin, albumin, uric acid, calcium × phosphate product (Ca × P product), and intact parathyroid hormone (iPTH) measurements], and duration of dialysis session were assessed for further analysis.

Patients' CBC was calculated by automated hematology analyzers. Leukopenia or thrombocytopenia is usually described as total WBC or PLT counts that are 2 standard deviations below the mean. Leukopenia was defined as WBC <4,000/μl, and thrombocytopenia was defined as PLT <100 × 10^3^/μl in this study (21). Parathyroidectomy was indicated if patients had an iPTH level >800 pg/ml with failure to vitamin D therapy. iPTH level was determined using a chemiluminescence immunoassay (CLIA, Immulite 2000) (22). CBC was checked at least every 3 months. Ferritin, iPTH, and clearance of urea to the volume of distribution (*Kt/V*) were examined every 6 months. Single-pool *Kt*/*V* was determined using 2-point urea remodeling with the Daugirdas equation: single-pool *Kt*/*V* = –ln[(1–urea reduction ratio)−0.008 × session length]–[4–3.5 × (1–urea reduction ratio)] × ultrafiltration/postdialysis weight (23). Chronic liver disease was defined as a persistent inflammatory condition of the liver in which biochemical and imaging abnormalities were present over a period of 6 months (24, 25). The following disorders were defined as rheumatic diseases: rheumatoid arthritis, systemic lupus erythematosus, Sjogren's syndrome, and spondyloarthropathies.

In our research, we determined that leukopenia or thrombocytopenia that continued for more than 6 months was defined as chronic leukopenia or thrombocytopenia. Patients who had leukopenia or thrombocytopenia at the initiation of ESRD but had normal CBC (WBC ≥4,000/μl or PLT ≥100 × 10^3^/μl) within 6 months after HD were classified as having transient leukopenia or thrombocytopenia.

### Statistical Analysis

The baseline characteristics of the enrolled patients in our study are displayed as the total number (*n*) and proportion (%). Pearson's chi-square test was used to compare categorical variables. Adjusted hazard ratios (aHRs) and 95% confidence intervals (CIs) were examined using the Cox proportional hazards model. Risk factors with *P* < 0.1 in the univariate model were selected for further evaluation in the multivariate analysis. The cumulative incidence of chronic leukopenia and thrombocytopenia was illustrated by means of the Kaplan–Meier method. Data management and statistical analysis were carried out using R software, version 3.5.2 (R Foundation for Statistical Computing, Vienna, Austria).

## Results

### Clinical Characteristics of the Study Population

**Figure 1:**
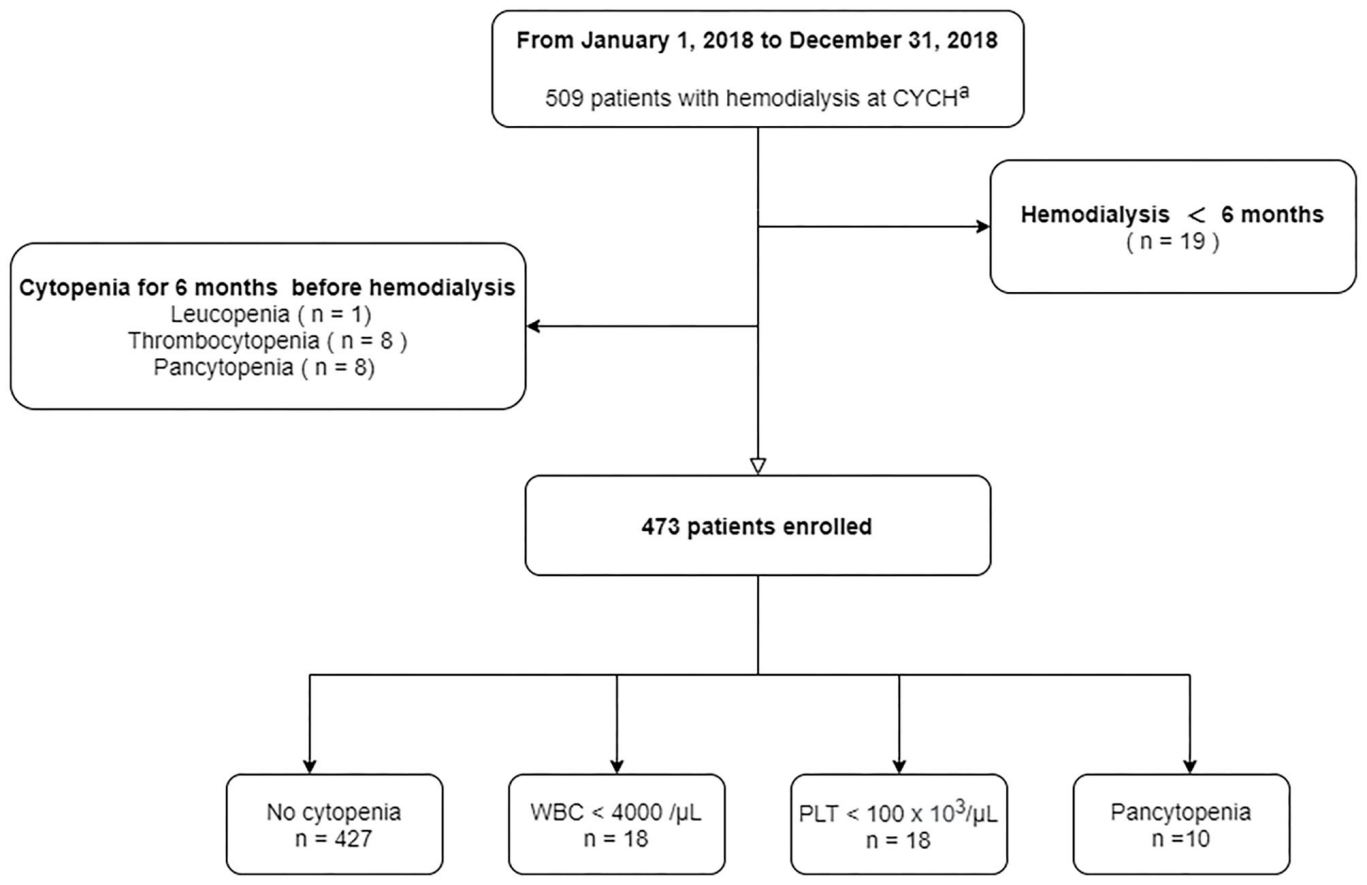
Patient selection. ^*a*^CYCH, Ditmanson Medical Foundation Chia-Yi Christian Hospital.

At the end of our study period, a total of 509 patients, including three with post kidney transplantation, with HD were identified at the Ditmanson Medical Foundation Chia-Yi Christian Hospital. Nineteen patients in our cohort were followed up for <6 months. Patients who had chronic leukopenia (*n* = 1), chronic thrombocytopenia (*n* = 8), or pancytopenia (*n* = 8) before dialysis therapy were excluded (Figure 1). Ultimately, 473 patients (55% male and 45% female) were enrolled in the final cohort. The median age of our patients at the end of follow-up was 64.6 years, with the interquartile range (IQR) from 57.0 to 72.4. Patients were followed up for a median of 5.7 years.

### Transient Leukopenia and Transient Thrombocytopenia

In this study, we found 18 (3.8%) and 24 (5%) patients with transient leukopenia and transient thrombocytopenia, respectively. All of them had WBC ≥4,000/μl and PLT ≥100 × 10^3^/μl within 6 months after HD. Nevertheless, we did not identify any risk factors or biomarkers to predict transient leukopenia and transient thrombocytopenia based on the univariate analysis (Supplementary Table 1).

**Figure 2:**
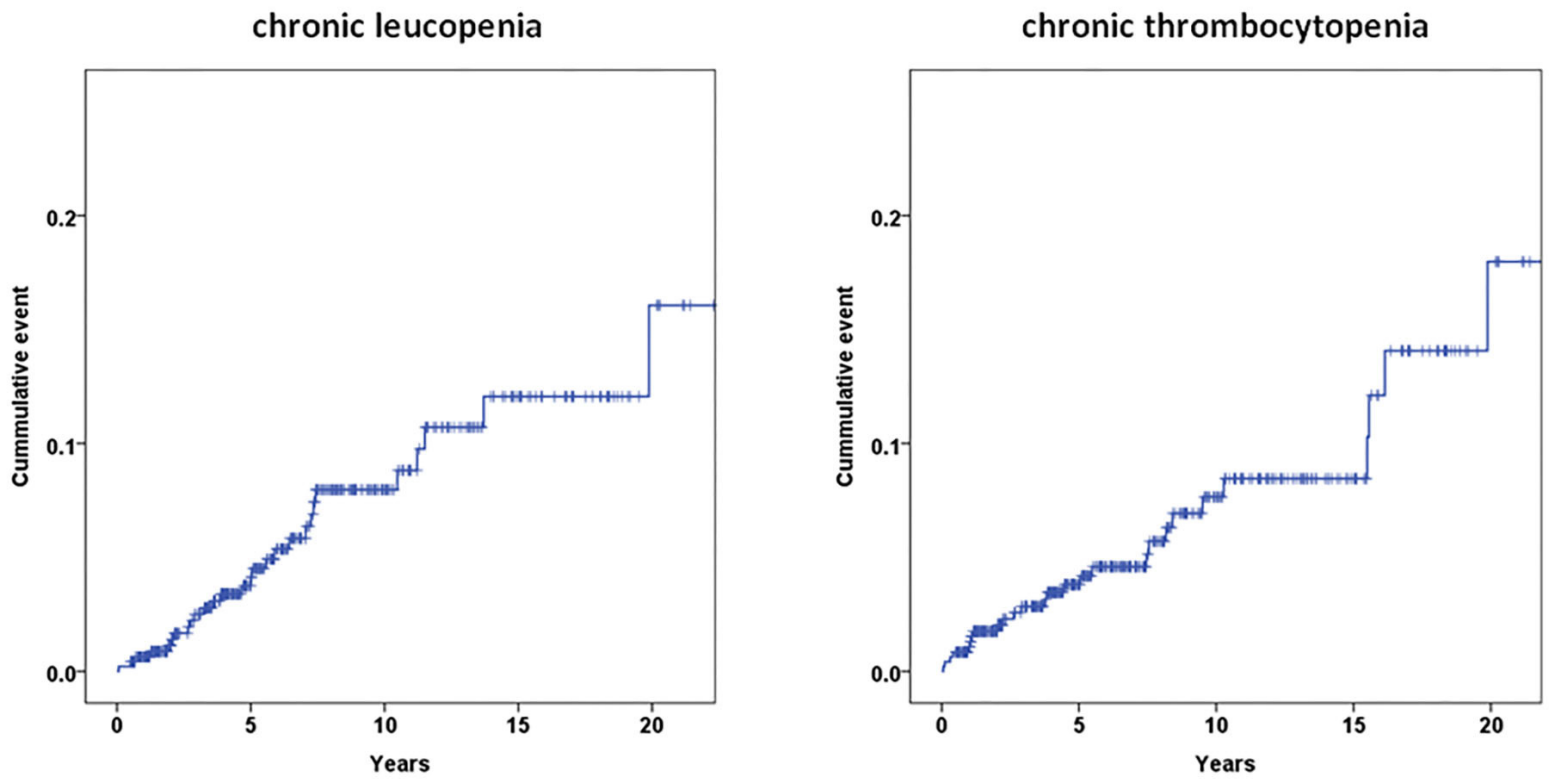
Cumulative incidence of chronic leukopenia and chronic thrombocytopenia.

**Figure 3:**
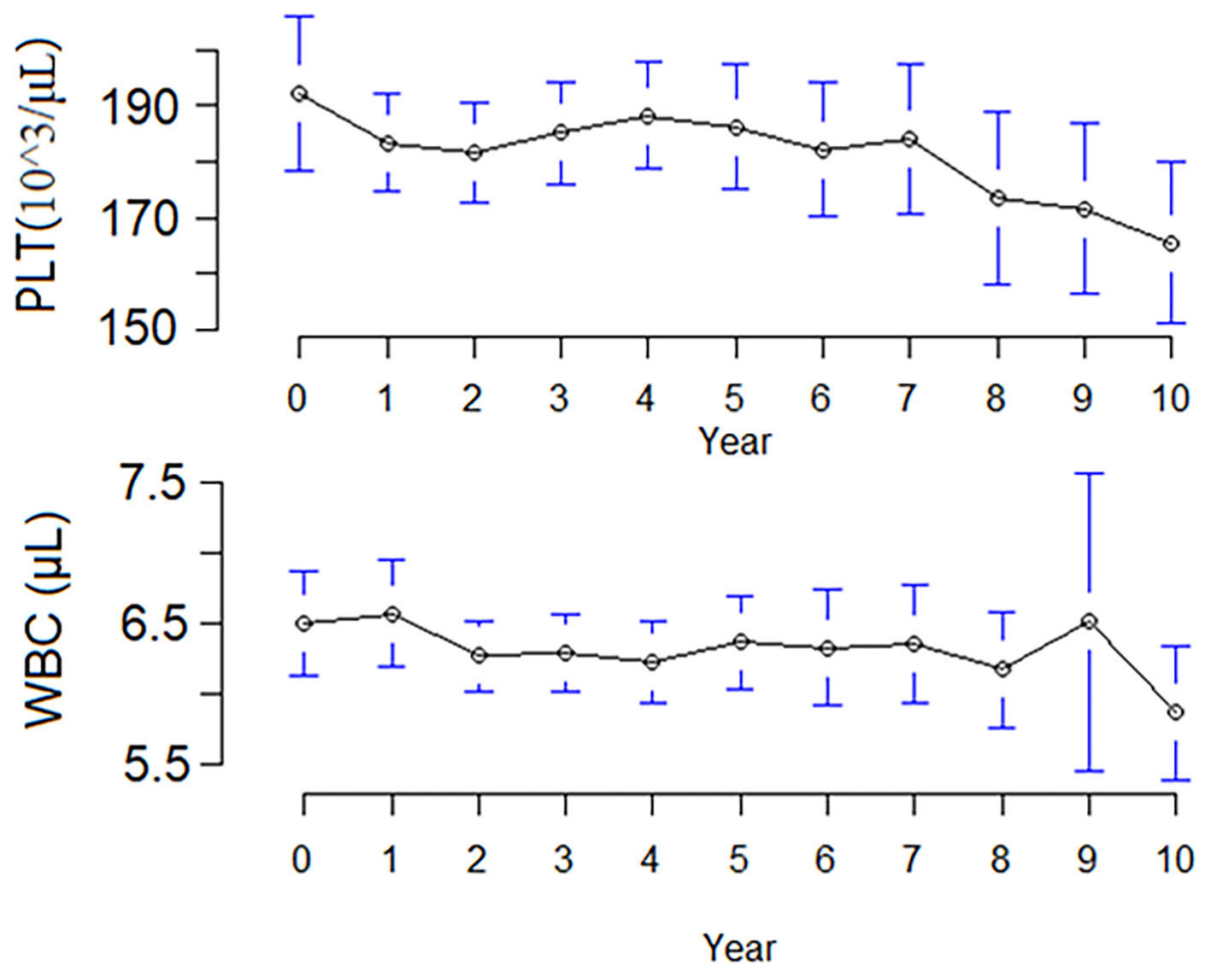
The trend (mean) of patients' white blood cell and platelet counts after hemodialysis.

At the end of follow-up, 46 (9.7%) patients had a hematologic abnormality, including 18 (3.8%) with chronic leukopenia, 18 (3.8%) with chronic thrombocytopenia, and 10 (2.1%) with pancytopenia. The incidence of chronic leukopenia and thrombocytopenia was 5.1 cases per 1,000 individuals. The median times to leukopenia and thrombocytopenia were 4.8 (IQR, 2.5–7.2) and 3.8 (IQR, 1.1–8.9) years. The cumulative incidence curve of leukopenia and thrombocytopenia in HD patients is displayed in Figure 2. The clinical characteristics of chronic leukopenia, chronic thrombocytopenia, and pancytopenia patients are summarized in Table 1. In this cohort, the PLT count and WBC count trends decreased with time (Figure 3).

**Table 1:** Characteristics of patients with hemodialysis (*n* = 473).

**Characteristics**	Total^*^n = 473	**Leukopenia *n* = 18**	**Thrombocytopenia *n* = 18**	**Both *n* = 10**	***P*-value**
	***n* (%)**	***n* (%)**	***n* (%)**	***n* (%)**	
Median age, years (IQR) at last F/U	64.6 (57.1–72.5)	64.0 (54.0–71.8)	67.0 (59.9–79.1)	65.4 (58.9–78.1)	0.632
≥65	233 (49.2)	10 (55.6)	9 (50)	5 (50)	0.991
<65	240 (50.8)	8 (44.4)	9 (50)	5 (50)	
Median age, years (IQR) at HD	58.3 (48.2–67.1)	57.5 (42.2–63.0)	59.3 (46.2–71.9)	56.6 (44.8–70.6)	0.859
≥60	211 (44.6)	8 (44.4)	9 (50)	5 (50)	0.935
<60	262 (55.4)	10 (55.6)	9 (50)	5 (50)	
Duration of hemodialysis, years (IQR)	5.7 (3.0–10.3)	7.4 (5.4–12.0)	6.7 (4.0–11.7)	9.9 (6.0–15.4)	0.006
Time to event, years (IQR)	–	5.1 (2.7–7.4)	–	4.4 (2.2–5.6)	
Time to event, years (IQR)	–	–	3.7 (1.0–9.1)	6.2 (2.1–8.1)	
BMI (IQR)	22.4 (20.2–25.7)	21.4 (19.8–24.6)	24.2 (22.0–25.7)	19.6 (17.3–21.0)	0.046
**Gender**					
Female	213 (45)	10 (55.5)	6 (33.3)	5 (50)	0.609
Male	260 (55)	8 (44.5)	12 (66.7)	5 (50)	
**Comorbidities**					
Chronic hepatitis C	79 (17.1)	2 (11.1)	5 (27.7)	4 (40)	0.124
Chronic hepatitis B	60 (13)	2 (11.1)	2 (11.1)	2 (20)	0.877
Chronic liver disease	69 (14.5)	1 (5.8)	6 (33.3)	5 (50)	0.001
Rheumatologic disease	26 (5.5)	1 (5.8)	0	0	0.836
Diabetes mellitus	248 (52.5)	5 (29.4)	9 (50)	3 (30)	0.104
Cerebral vascular disease	65 (13.7)	1 (5.8)	2 (11.1)	1 (10)	0.924
Hypertension	432 (91.5)	16 (94.1)	15 (83.3)	8 (80)	0.196
Cancer	71 (15.0)	2 (11.1)	4 (22.2)	1 (10)	0.776
Urothelial carcinoma	28 (5.9)	1 (5.5)	2 (11.1)	0	0.597
Hepatocellular carcinoma	9 (1.9)	1 (5.5)	1 (5.5)	0	0.576
Breast cancer	8 (1.7)	0	0	0	1.000
Others	26 (5.4)	0	1 (5.5)	1 (10)	0.693
**Cancer treatment**					
Surgical resection	59 (12.4)	2 (11.1)	4 (22.2)	1 (10)	0.648
Chemotherapy	23 (5.4)	1 (5.5)	2 (11.1)	0	0.561
Radiotherapy	10 (2.1)	0	1 (5.5)	0	0.654
**Parameter**					
P (mg/dl, IQR)	5.1 (4.2–6.1)	5.2 (4.5–5.9)	4.6 (4.0–5.8)	4.4 (4.3–4.7)	0.311
Ferritin (ng/ml, IQR)	500 (342–652)	487 (239–585)	615 (426–912)	546 (499–829)	0.153
Albumin (g/dl, IQR)	3.9 (3.7–4.1)	4.0 (3.7–4.1)	3.9 (3.6–4.0)	3.8 (3.7–4.1)	0.523
Ca × P product	47.0 (37.7–56.5)	47.9 (41.4–55.6)	42.3 (31.2–53.0)	41.8 (40.4–42.7)	0.237
iPTH^*^ (pg/ml, IQR)	317 (129–696)	455 (260–937)	400 (219–776)	451 (138–862)	0.446
Ca × P product >55	127 (28.2)	4 (22.2)	7 (38.8)	3 (30)	0.613
Parathyroidectomy	84 (18.5)	6 (33.3)	5 (29.4)	2 (20)	0.250
^*^iPTH <240 pg/ml	178 (56.1)	6 (54.5)	7 (70)	4 (80)	0.611
^*^iPTH <300 pg/ml	155 (48.8)	4 (36.3)	7 (70)	3 (60)	0.430
^*^iPTH <600 pg/ml	112 (35.3)	3 (17.6)	5 (38.4)	2 (42.8)	0.486
Ferritin >800 ng/ml	49 (11.9)	2 (11.7)	4 (16.6)	3 (33.3)	0.047
Kt/V >1.2	424 (90.2)	16 (88.9)	15 (83.3)	8 (80)	0.276
P >5 mg/dl	241 (52.8)	10 (55.5)	7 (41.1)	2 (22.2)	0.215
Uric acid >7 mg/dl	228 (55.3)	12 (70.5)	11 (73.3)	4 (44.4)	0.246
Vitamin D supplementation	63 (13.7)	3 (16.6)	1 (5.8)	0	0.553
Iron supplementation	163 (35.5)	7 (38.8)	7 (41.1)	3 (30)	0.919
Erythropoiesis-stimulating agents	435 (94.9)	18 (100)	17 (100)	9 (90)	0.594
Hemodialysis access					0.319
A-V fistula	279 (69.9)	14 (87.5)	12 (75)	6 (66.6)	
A-V graft	97 (24.3)	1 (6.2)	2 (12.5)	3 (33.3)	
Catheter	23 (5.7)	1 (6.2)	2 (12.5)	0	

IQR, interquartile range; F/U, follow-up; HD, hemodialysis; BMI, body mass index.

* *Excluding patients with parathyroidectomy*.

### Risk Factors for Chronic Leukopenia and Thrombocytopenia

In the univariate analysis, an age of ≥60 years at the initiation of HD, serum ferritin levels >1,000 ng/ml, and transient leukopenia were associated with chronic leukopenia. Age at the initiation of HD, HCV infection, liver parenchymal disease, serum ferritin levels >800 ng/ml, *Kt/V* <1.2, vitamin D from IV supplementation, serum phosphorous level, parathyroidectomy, and transient thrombocytopenia were risk factors for chronic thrombocytopenia (Table 2). In the multivariate Cox analysis, age ≥60 years at the initiation of HD (aHR, 2.71; 95% CI, 1.06–6.89, *P* = 0.036) was an independent risk factor of chronic leukopenia. Conversely, an age ≥60 years at the initiation of HD (aHR, 2.83; 95% CI, 1.08–7.35, *P* = 0.032), liver parenchymal disease (aHR, 3.31; 95% CI, 1.27–8.61, *P* = 0.013), and serum ferritin levels >800 mg/dl (aHR, 3.29; 95% CI, 1.29–8.39, *P* = 0.012) were predictors for chronic thrombocytopenia (Table 2). Additionally, we found patients with vitamin D from IV supplementation (aHR, 0.13; 95% CI, 0.01–1.16, *P* = 0.066) and higher serum phosphorous level (aHR, 0.73; 95% CI, 0.53–1.02, *P* = 0.068) were prone to have a lower risk of chronic thrombocytopenia (Table 2). Finally, cancer type, strategy of cancer treatment, and HD access were not risk factors (Supplementary Table 2).

**Table 2:** Risk factors for patients with chronic leukopenia and chronic thrombocytopenia.

	**Chronic leukopenia**		**Chronic thrombocytopenia**	
	**HR (95% CI)^**a**^^b^**	***P*-value**	**HR (95% CI)^**a**^^b^**	***P*-value**
Age ≥60 years (at HD)	2.71 (1.06–6.89)	0.036	2.83 (1.08–7.35)	0.032
Chronic liver disease	0.96 (1.03–3.39)	0.954	3.31 (1.27–8.61)	0.013
Ferritin >800 ng/ml	–	–	3.29 (1.29–8.39)	0.012
Ferritin >1,000 ng/ml	2.61 (0.72–9.45)	0.141	–	–
Transient thrombocytopenia at HD^*^	1.37 (0.37–5.01)	0.629	3.91 (1.70–8.97)	0.001
P mg/dl (continuous)			0.73 (0.53–1.02)	0.066
Vitamin D supplementation			0.13 (0.01–1.16)	0.068
HCV			0.76 (0.27–2.11)	0.606
Parathyroidectomy			0.56 (0.16–1.90)	0.357
*Kt/V* ≥ 1.2			0.62 (0.19–2.01)	0.431

HR, hazard ratio; CI, confidence interval; HD, hemodialysis; HCV, hepatitis C virus; BMI, body mass index.

a Treatment was analyzed as a time-dependent covariate in the Cox regression model.

b Adjusted for gender, age at HD, BMI, parathyroidectomy, and chronic liver disease.

* *PLT <100 × 10^3^/μl at the beginning of HD. Return to normal range within 6 months*.

## Discussion

To the best of our knowledge, this is the first study to report that chronic leukopenia and thrombocytopenia are long-term complications of HD. We find that patient age above 60 years at the initiation of dialysis is a risk factor for both chronic leukopenia and thrombocytopenia. High serum ferritin levels and transient thrombocytopenia are risk factors for chronic thrombocytopenia. Furthermore, vitamin D from IV supplementation and serum phosphorous levels have been shown to be associated with chronic thrombocytopenia. Cancer type and cancer treatment were not associated with cytopenia. In spite of lacking direct evidence, we suppose that HD may play a role in chronic cytopenia.

Transient leukopenia during HD has been well-described and is usually associated with hypersensitivity reactions to dialyzer membranes and the activation of the complement cascade pathway, which leads to the pulmonary sequestration of neutrophils (12, 26, 27). Several studies have shown that the presence of leukemia during the initiation of HD can predict mortality (28, 29); however, the association between transient leukopenia and patients' outcome is unknown (30). In the present study, we identified 3.8% of patients had transient leukopenia. Additionally, HCV infection may relate to leukopenia and/or thrombocytopenia in HD patients. A retrospective study conducted by Ng et al. (31) reported that 11 out of 28 HD patients who were anti-HCV-positive had leukopenia, and the odds ratio (OR) was 6.22. Nevertheless, HCV infection was insignificant in our study. The possible reason contributing to this discrepancy is unknown.

Compared to healthy populations, reduced PLT counts in predialysis and HD patients have been observed (13, 32). The effect of dialysis on PLT count is multiple; for example, some patients start HD in critical condition and the development of thrombocytopenia is not uncommon (33). Some medications, such as antibiotics used in acute sepsis, are suspected to suppress bone marrow function (34, 35). Heparin, which is usually used for extracorporeal circuit anticoagulation, has the potential to induce thrombocytopenia (13, 36). Consumption of platelets may be attributed to either an intravascular graft or dialysis catheter, thrombotic microangiopathy caused by hypertensive crisis, or thrombotic thrombocytopenic purpura (37, 38). In addition, dialyzer membranes also initiate the complement cascade pathway, platelet adhesion, aggregation, and activation leading to thrombocytopenia (13, 39, 40). Ng et al. (31) also demonstrated that HCV infection was a risk factor (OR = 3.27) for thrombocytopenia in dialysis patients. In our research, we used a lower cutoff point for the PLT count (<100 × 10^3^/μl) to define thrombocytopenia, and the OR of HCV infection was 2.14 with a borderline significance.

Chronic leukopenia and thrombocytopenia are rarely described in dialysis patients. The present study discloses that hematologic abnormalities are long-term complications of dialysis. One possible etiology is secondary hyperparathyroidism and renal osteodystrophy, which are long-term complications of dialysis. Here, we found vitamin D from IV supplementation, a medical treatment for dialysis related to hyperparathyroidism, is a risk factor for chronic thrombocytopenia (41). Both primary and secondary hyperparathyroidism are known causes of secondary myelofibrosis (8, 10, 16, 17). Reversal of bone marrow fibrosis has been demonstrated after parathyroidectomy (42). However, parathyroidectomy, a surgical intervention to control hyperparathyroidism, in our cohort was insignificant and may be ascribed to limited cases. Additionally, chronic inflammation in ESRD is another possible mechanism. It is well-known that aberrant inflammation signals impair hematopoietic stem cell self-renewal and the function of the bone marrow (43). Serum ferritin is a marker of chronic inflammation (44); we observed that high serum ferritin levels are associated with cytopenia. Ferritin levels are usually correlated with inflammatory activity (45). Recently, chronic innate immune signaling and ineffective hematopoiesis have been established (46). Basiorka et al. (47) reported that activation of the NLRP3 inflammasome contributed to hematopoietic stem cell death and led to myelodysplastic syndromes. Moreover, crystal deposition in bone marrow may be rare but has an adverse impact on hematopoiesis. For example, Sharma et al. (18) described bone marrow oxalate deposition in two patients with systemic oxalosis and ESRD. Ananthanarayanan and Kini (48) presented a case of refractory thrombocytopenia receiving a bone marrow biopsy, and gout tophi were observed in the bone marrow.

This study had some limitations. First of all, we lacked bone marrow data and cytogenetic analysis to clarify the etiology of leukopenia or thrombocytopenia, such as myelodysplastic syndrome or acute leukemia. Potential confounding factors, such as exposure to cytotoxic agents or chemicals in the workplace and lifestyle variations, were not completely available for our cohort. Patients were possibly exposed to bacterial endotoxin during the HD sessions, but the endotoxin level was not recorded in our patients. Second, the laboratory data, including WBC, PLT, iPTH, ferritin, and electrolytes, were dynamic. For instance, patients with active sepsis may have had hyperferritinemia and thrombocytopenia. Potential confounding factors, such as exposure to cytotoxic agents and lifestyle variations, were not completely available for our cohort. Third, it is a small, single-center, retrospective cohort analysis of HD patients. Lastly, damage of hematopoietic stem cells is a continuous process (49), and it takes time for an abnormal hematological profile to develop. Generally, the adjusted 3- and 5-year survival rates were reported to be 70% and 50% in ESRD patients without kidney transplantation, respectively (2, 3). The median time to cytopenia in our patients was around 4 years, suggesting that mortality is an important competing factor. Thereafter, further prospective studies are needed to validate our findings.

In conclusion, our study indicates that hematological abnormality is a long-term complication of HD. Old age was a risk factor for chronic leukopenia. The risk of chronic thrombocytopenia included patients' age at the initiation of HD, serum ferritin levels >800 mg/dl, and transient thrombocytopenia. Finally, the role of cytopenia on uremic prognosis and the impact of uremic toxins on hematopoietic stem cells are worth investigating to find out the possible mechanism and to improve patients' quality of life.

## Data Availability Statement

The datasets generated during and/or analyzed during the current study are available from the corresponding author on reasonable request. Requests to access these datasets should be directed to dtmedg3@yahoo.com.tw.

## Ethics Statement

This study was conducted in concordance with institutional patient safety laws and has been approved by the Institutional Review Board of Chiayi Christian Hospital (approval no. CYCH-IRB-2019042). This study was performed in accordance with the Declaration of Helsinki. Written informed consent for participation was not required for this study in accordance with the national legislation and the institutional requirements.

## Author Contributions

Y-TL and P-HH contributed to protocol/project development, contributed to data collection or management, and contributed to manuscript writing/editing. Y-TL contributed to data analysis. W-YW, C-HK, M-YL, Y-CL, Y-HH, and P-HH contributed to manuscript review. Y-HH and P-HH were the scientific advisers. All authors participated in the interpretation of the studies and analysis of the data and reviewed and approved the final version of the manuscript.

## Conflict of Interest

The authors declare that the research was conducted in the absence of any commercial or financial relationships that could be construed as a potential conflict of interest.
