# Supplementary material for: Older Age and High Serum Ferritin Levels Associated With the Risk of Chronic Cytopenia in Hemodialysis Patients
Source: Front Med (Lausanne). 2020 Oct 29;7:568350. doi: 10.3389/fmed.2020.568350 (PMC7662880; doi:10.3389/fmed.2020.568350)
Supplement: Supplementary file 1 [file Data_Sheet_1.doc]

| **Supplemental Table 1. Risk factors for Patients with Transient Leucopenia and Transient Thrombocytopenia** | | | | |
| --- | --- | --- | --- | --- |
| Predictive variables | Transient leucopenia | | Transient thrombocytopenia | |
|  | OR (95% CI) | *P* value | OR (95% CI) | *P* value |
| Age ≥ 60 (at HD) | 0.55 (0.18-1.49) | 0.271 | 0.66 (0.26-1.56) | 0.332 |
| Male Gender | 0.93 (0.35-2.47) | 1.000 | 1.22 (0.52-2.97) | 0.695 |
| Chronic Hepatitis C | 1.99 (0.60-5.71) | 0.228 | 2.14 (0.77-5.42) | 0.108 |
| Chronic Hepatitis B | 1.58 (0.37-5.16) | 0.501 | 0.81 (0.15-2.82) | 1.000 |
| Chronic liver disease | 1.79 (0.49-5.35) | 0.344 | 1.67 (0.52-4.51) | 0.271 |
| Autoimmune disease | 3.54 (0.61-14.0) | 0.079 | 1.59 (0.17-7.35) | 0.634 |
| iPTH > 200 ng/ml | 1.18 (0.10-61.5) | 1.000 | 2.16 (0.25-103) | 0.676 |
| P > 5.5 mg/dl | 0.78 (0.26-2.46) | 0.616 | 0.74 (0.27-2.17) | 0.625 |
| Ca > 8.4 mg/dl | 1.61 (0.55-4.85) | 0.339 | 1.78 (0.63-5.28) | 0.240 |
| OR, odd ratio ratio; CI, confidence interval | | | | |

| **Supplemental Table 2 Univariate analysis for Chronic Leucopenia and Chronic Thrombocytopenia** | | | | |
| --- | --- | --- | --- | --- |
| **Predictive variables** | **Chronic Leucopenia** | | **Chronic Thrombocytopenia** | |
|  | **HR (95% CI)^a^** | ***P* value** | **HR (95% CI)^a^** | ***P* value** |
| **Age ≥ 60 (at HD)** | 2.12 (0.97-4.63) | 0.057 | 3.01 (1.31-6.96) | 0.009 |
| **Sex (male)** | 0.84 (0.40-1.78) | 0.666 | 1.59 (0.74-3.41) | 0.231 |
| **BMI (Comt.)** | 0.93 (0.84-1.04) | 0.203 | 0.98 (0.90-1.09) | 0.832 |
| **HD access** |  |  |  |  |
| A-V fistula | reference |  | reference |  |
| A-V graft | 0.23 (0.03-1.82) | 0.167 | 1.79 (0.68-4.65) | 0.232 |
| Catheter | 1.11 (0.14-8.48) | 0.917 | 1.33 (0.30-5.90) | 0.704 |
| **Comorbidities** |  |  |  |  |
| HCV | 1.27 (0.51-3.15) | 0.601 | 2.22 (1.00-4.95) | 0.049 |
| HBV | 1.13 (0.39-3.27) | 0.817 | 1.04 (0.36-3.03) | 0.930 |
| Liver disease | 1.74 (0.70-4.32) | 0.226 | 4.30 (2.01-9.19) | <0.001 |
| Rheumatologic disease | 0.47 (0.06-3.5) | 0.464 | - | - |
| Diabetes mellitus | 0.57 (0.24-1.34) | 0.198 | 1.26 (0.56-2.82) | 0.569 |
| Cerebral vascular disease | 0.52 (0.12-2.21) | 0.377 | 0.74 (0.22-2.48) | 0.633 |
| Hypertension | 1.21 (0.36-4.09) | 0.754 | 0.79 (0.29-2.14) | 0.643 |
| Cancer | 0.74 (0.25-2.14) | 0.578 | 1.35 (0.57-3.20) | 0.485 |
| Chemotherapy | 0.58 (0.07-4.33) | 0.601 | 1.19 (0.28-14.7) | 0.501 |
| Radiotherapy | 1.08 (0-**) | 0.996 | 1.61 (0.21-12) | 0.640 |
| Surgical resection | 0.72 (0.21-2.39) | 0.592 | 1.25 (0.47-3.3) | 0.651 |
| **Parameter** |  |  |  |  |
| CaxP > 55 | 0.98 (0.41-2.34) | 0.975 | 1.78 (0.81-3.92) | 0.148 |
| Parathyroidectomy | 0.63 (0.26-1.5) | 0.298 | 0.44 (0.17-1.14) | 0.091 |
| iPTH < 240^b^ | 1.00 (0.30-3.32) | 0.994 | 2.16 (0.68-6.81) | 0.188 |
| iPTH < 800^b^ | 0.47 (0.06-3.75) | 0.482 | 0.66 (0.15-2.96) | 0.595 |
| Ferritin > 800 | 2.13 (0.80-5.68) | 0.128 | 3.67 (1.52-8.91) | 0.003 |
| Ferritin > 1000 | 2.84 (0.97-8.28) | 0.055 | 4.71 (1.87-11.9) | 0.001 |
| Kt/V > 1.2 | 0.61 (0.21-1.79) | 0.374 | 0.43 (0.16-1.14) | 0.088 |
| P (cont) | 0.98 (0.76-1.27) | 0.903 | 0.73 (0.54-0.98) | 0.040 |
| Pi > 5 | 0.79 (0.37-1.71) | 0.562 | 0.53 (0.23-1.19) | 0.125 |
| Uric acid >7 | 1.34 (0.61-2.98) | 0.458 | 1.43 (0.62-3.28) | 0.392 |
| Albumin (Cont.) | 1.34 (0.48-3.73) | 0.566 | 0.56 (0.22-1.41) | 0.219 |
| Vit. D supplementation | 0.57 (0.17-1.91) | 0.368 | 0.19 (0.02-1.43) | 0.107 |
| Iron Supplementation | 1.17 (0.54-2.55) | 0.678 | 1.27 (0.58-2.78) | 0.548 |
| EPO supplementation | 1.85 (0.25-13.6) | 0.545 | 1.98 (0.26-14.7) | 0.501 |
| Transient leucopenia at HD^**^ | 2.64 (0.78-8.95) | 0.117 | 2.56 (0.75-8.72) | 0.131 |
| Transient thrombocytopenia at HD^***^ | 2.02 (0.99-4.13) | 0.051 | 3.40 (1.60-7.25) | 0.001 |
| HR, hazard ratio; CI, confidence interval;  ^a^Treatment was analyzed as a time-dependent covariate in the Cox regression model.  ^b^Excluding patients with parathyroidectomy  *Don’t converge  ** WBC < 4000 at HD  *** PLT < 100 at HD | | | | |
